# Effect of heterologous bone marrow mononuclear cell transplantation on midpalatal expansion in rats

**DOI:** 10.3892/etm.2015.2253

**Published:** 2015-02-03

**Authors:** JIE GUO, LUE WANG, HAIHUA XU, XIAOXIA CHE

**Affiliations:** 1 Department of Orthodontics, School of Stomatology, Shandong University, Jinan, Shandong 250012, P.R. China; 2 Shandong Provinicial Key Laboratory of Oral Biomedicine, Shandong University, Jinan, Shandong 250012, P.R. China; 3 College of Life Science and Technology, Beijing University of Chemical Technology, Beijing 100029, P.R. China; 4 Department of Orthodontics, School of Stomatology, Capital Medical University, Beijing 100050, P.R. China

**Keywords:** midpalatal expansion, bone marrow mononuclear cell, cell transplantation, receptor activator of nuclear factor κB ligand/osteoprotegerin

## Abstract

The aim of this study was to explore whether bone marrow mononuclear cell (BMMC) transplantation is able to accelerate the bone remodeling induced by midpalatal expansion in rats. A total of 48 male Sprague-Dawley rats (mean weight, 208.36±7.32 g) were divided into control and midpalatal expansion with or without BMMC transplantation groups. Histological and morphological changes were observed in each group. The osteogenic activities and differential potentials of the transplanted BMMCs labeled with bromodeoxyuridine in the midpalatal bone tissue were assessed by osteocalcin expression. The receptor activator of nuclear factor κB ligand (RANKL)/osteoprotegerin (OPG) ratio was determined by reverse transcription-quantitative polymerase chain reaction (RT-qPCR) to reflect the equilibrium between bone resorption and formation. The results demonstrated that the width of the maxillary dental arch increased distinctly within 2 weeks of midpalatal expansion with BMMC transplantation. The morphology of the midpalatal suture in this group changed significantly; the cartilage was completely replaced by fibrous-like tissue expressing osteocalcin. The palatal bone was reorganized from a cancellous form into a mature compact structure after an additional 2-week relapse period. Immunostaining results indicated that the heterologous transplanted BMMCs survived and differentiated into osteoblasts during the remodeling induced by midpalatal expansion. The RANKL/OPG expression ratio significantly decreased after 2 weeks of midpalatal expansion with BMMC transplantation due to the inhibition of RANKL expression. Heterologous BMMC transplantation appears to accelerate the midpalatal bone remodeling induced by expansion of the rats through increasing the number of osteoprogenitor cells and regulating the RANKL-OPG signaling pathway.

## Introduction

Rapid midpalatal expansion (RME) is a common treatment for patients with a narrow maxillary dental arch. It has been effectively used to correct transverse maxillary discrepancies in children and adolescents up to the pubertal stage (1). The maxillary dental arch increases rapidly with active tissue remodeling in the palate (2). Surgical assistance, such as measured tipping of the anchor teeth, is often used to eliminate side-effects (3). Favorable orthopedic responses can be achieved for patients prior to and/or during pubertal growth (4,5). Certain clinicians also use expansion treatments to treat adult patients who have a narrow maxillary arch with increasing skeletal resistance (6,7). Although the effectiveness of this treatment has been reported, the long-term dental and skeletal stability remains uncertain (8).

Retention appliances are typically used to maintain the results of tooth movement until the architectural environment achieves equilibrium. However, even following the retention period, the expanded maxillary dental arch has a strong tendency to rebound to its previous form (9). A greater relapse tendency has been reported for midpalatal expansion in adults due to the decreasing rate of bone regeneration. Orthodontists and biologists have studied the mechanism of stretch-mediated osteogenesis in the expanded suture for decades; however, relapse continues to be unavoidable (10).

Accelerating bone formation during midpalatal expansion would be beneficial in preventing relapse and shortening the retention period. Oztürk *et al* (11) found that zoledronic acid had positive effects on bone formation in response to expansion; it was able to decrease the relapse rate following expansion in rats. Transforming growth factor-β1 also been reported to stimulate bone formation in the expanding suture (12). Results of recent animal studies have demonstrated the potential utility of stem cell transplantation for skeletal regeneration (13–15). Stem cell transplantation may also shed light on midpalatal expansion in adult patients as the impaired bone formation activity can be attributed to the partial (16) or reduced osteo-formation capabilities of osteoprogenitor cells in aged individuals (17).

In the present study, a rat midpalatal expansion model was used to elucidate the mechanism underlying bone remodeling and determine whether bone marrow cell transplantation was able to accelerate it.

## Materials and methods

### Animals and grouping

A total of 48 male Sprague-Dawley (SD) rats (Vital River Laboratory, Beijing, China) were used in this study. Their mean weight was 208.36±7.32 g. The animal protocol was approved by the Institutional Animal Care and Use Committee of Capital Medical University (Beijing, China).

Three rats that did not receive any intervention were observed as a blank control (BC) for histological morphology. The remaining rats were divided into five groups (n=9 per group): Non-expansion control group (NC), in which the midpalatal suture of the rats was cut; expansion group (Exp), in which the rats underwent midpalatal expansion for 2 weeks; expansion and transplantation group (EaT), in which rats underwent midpalatal expansion for 2 weeks with midpalatal incision and bone marrow mononuclear cell (BMMC) transplantation: expansion and relapse group (ExR); and expansion, transplantation and relapse group (EtR). In the two relapse groups, the rats underwent midpalatal expansion for 2 weeks with midpalatal incision without or with BMMC transplantation, respectively, and two weeks after the removal of the expansion appliance, the rats were sacrificed for the observation of palatal changes. Samples from six rats from each group were used for reverse transcription-quantitative polymerase chain reaction (RT-qPCR) analysis and the other three were used for histological and immunohistochemical assessment.

### Expansion of the midpalatal suture

**Figure 1:**
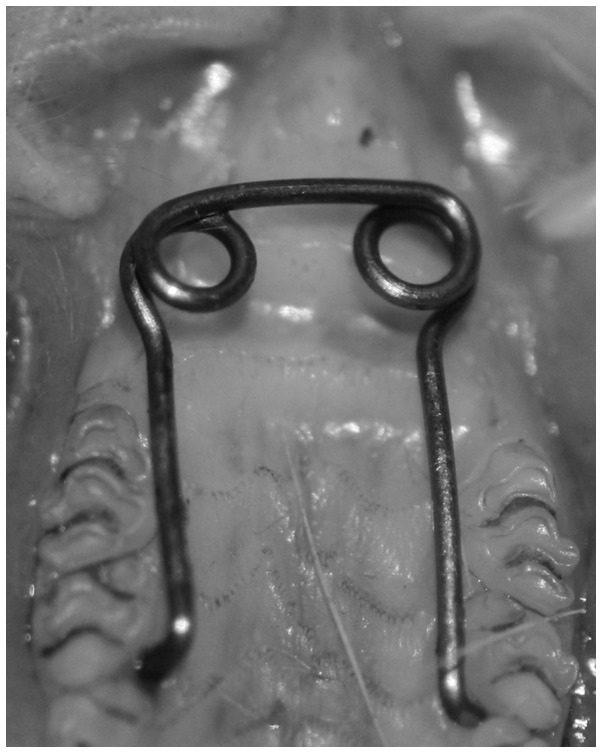
Midpalatal expansion appliance made by a 0.45-mm stainless steel wire.

The rats were subjected to midpalatal expansion as described in a previous study (18). The distal ends of the midpalatal expansion appliance (0.45 mm stainless steel A.J. Wilcock Australian Wire; A.J. Wilcock PTY., Ltd., Melbourne, Australia) were placed into the interproximate space between the second and third molars, and were activated through the ends of the compression helices to exert an initial expansion force of 150 g (Fig. 1). A 1.5-cm midsagittal incision was made anteroposteriorly. Expansion appliances were activated twice every other day to achieve maxillary expansion.

### BMMC culture and transplantation

Approximately 0.5 ml bone marrow aspirate was collected from the tibias of 2-month old BALB/C mice (Vital River Laboratory). The marrow cells were transferred to a 15-ml conical tube containing 1-ml Percoll (1.073 g/l; GE Healthcare, Piscataway, NJ, USA). The tube was centrifuged at 1,500 × g for 25 min. The mononuclear cells at the interface were collected, resuspended, then plated at 1.0×10^4^ cells/cm^2^ in Dulbecco’s modified Eagle’s medium (DMEM)/F12 medium supplemented with 20% fetal bovine serum (Gibco Life Technologies, Carlsbad, CA, USA) and antibiotics at 37°C in humid air with 5% CO_2_.

The cells were labeled with bromodeoxyuridine (BrdU; Sigma-Aldrich, St. Louis, MO, USA). Expanded and marked BMMCs (1×10^6^ cells/ml, 0.5 ml) were suspended in sterile medium, then were injected intra-orally into the masticatory muscle area opposite the first molar 2 days after the second activation of the expansion appliance; the expansion appliances were kept in place for 2 weeks. A 9% sodium chloride solution (5 mg/kg) was injected instead of labeled cells into the animals of the Exp and ExR groups.

### Posterior dentition arch width

To identify whether there was an expansion-induced widening effect of the midpalatal suture, three inter-molar distances of each rat were measured. This measurement was taken three times at the end of the experimental period to calculate the average increased posterior dentition width.

### Observation of histological changes

Dissected samples, including the midpalatal suture from the first to second molars of rats from all groups, were fixed in 10% formalin solution for 48 h. Tissue blocks were demineralized and embedded with paraffin routinely. Sections (5 μm) were cut and mounted on poly-L-lysine-coated glass slides. Cut sections were incubated at 60°C for 1 h, held in xylene and rehydrated through a series of ethanol solutions, then stained with hematoxylin and eosin for observation under a fluorescence microscope (Olympus BX61; Olympus, Tokyo, Japan).

### Osteogenic activities during midpalatal suture expansion

Osteocalcin expression indicated the commencement of active bone formation (18,19). Immunofluorescent staining for osteocalcin was performed. The paraffin sections were incubated with a polyclonal rabbit anti-osteocalcin antibody (1:100 dilution; cat. no. BA1677-1, Boster Biological Technology, Ltd., Wuhan, China) in a moist chamber at 4°C for 18 h, followed by tetramethyl-rhodamine-isothiocyanate-conjugated goat anti-rabbit IgG (1:50 dilution; cat. no. BA1090, Boster Biological Technology Co., Ltd.) at 37°C for 1 h. To visualize the cell nuclei in the palatal tissue, 4′,6-diamidino-2-phenylindole (Molecular Probes^®^, Life Technologies, Carlsbad, CA, USA) staining was used. Fluorescent images were captured using an Olympus BX61 fluorescence microscope with an excitation wavelength of 550 nm.

### Osteogenic capability of transplanted BMMCs

The transplanted BMMCs labeled with BrdU were identified and tracked by immunofluorescent staining with a mouse monoclonal antibody targeting BrdU (1:100 dilution; cat. no. B8434, Sigma-Aldrich). Double immunohistochemical staining for osteocalcin and BrdU was carried out using DouMax Vision™ (Maixin Biotechnology, Fuzhou, China), according to the manufacturer’s instructions, in order to confirm the osteogenic capability of the transplanted BMMCs in the palatal bone. The blue-black BrdU-positive areas were visualized with 5-bromo-4-chloro-3-indolyl-phosphate/nitro-blue-tetrazolium, whereas the salmon pink osteocalcin-positive areas were visualized with 3-amino-9-ethylcarbazole. Sections were counterstained with hematoxylin prior to mounting, and were visualized under an Olympus BX61 microscope.

### Receptor activator of nuclear factor κB ligand (RANKL) and osteoprotegerin (OPG) expression in midpalatal expansion

RANK, which is produced by osteoblasts, promotes osteoclastogenesis when it binds to its ligand RANKL. OPG acts as a decoy receptor, and can bind RANKL and prevent RANK signaling. The OPG-RANK-RANKL signaling pathway has been attributed a decisive role in the regulation of osteoblast and osteoclast activity to ensure normal bone turnover (20). In this experiment, RT-qPCR was performed to determine the expression of RANKL and OPG. The tissue blocks from the first to the second molars were harvested free of the overlying soft tissue. Specimens were powdered under liquid nitrogen and homogenized in TRIzol solution (Invitrogen Life Technologies, Carlsbad, CA, USA) using a homogenizer. The yield and purity of RNA were estimated spectrophotometrically using the ratio of absorbances at 260 and 280 nm (A260:A280). RNA was reverse transcribed to cDNA using the SuperScript reverse transcriptase system (Invitrogen Life Technologies) according to the manufacturer’s instructions. The primers used are listed in Table I. The RT-qPCR processes were run on the 7300 RT PCR detection system (Applied Biosystems, Foster City, CA, USA), and the results were analyzed using the software supplied with the system. The running conditions were incubation at 50°C for 2 min and 95°C for 10 min, followed by 60 cycles of incubation at 94°C for 15 sec and 60°C for 1 min. β-actin was used as an internal control. The expression levels of the target genes were correlated with β-actin using the 2^−ΔΔCt^ method. The RANKL/OPG ratio was calculated to reflect the equilibrium between bone resorption and formation.

### Statistical analysis

Data are expressed as mean ± standard deviation. The data were subjected to Fisher’s protected least significant difference test and one-way analysis of variance. Statistical analyses were performed using SPSS version 17.0 software (SPSS, Inc., Chicago, IL, USA). P<0.05 was considered to indicate a statistically significant difference.

## Results

### General condition of the rats

None of the rats in these groups experienced a significant loss of body weight during the experiment. Their food intake was disturbed during rapid expansion of the suture at the beginning of the experiment; however, the body weights recovered afterward.

### Expanded posterior dentition arch width in the EaT group

The posterior dentition arch width values for each group were as follows: NC, 7.44±0.29 mm; Exp, 7.55±0.18 mm; EaT, 8.37±0.32 mm; ExR 7.33±0.21 mm; and EtR 7.69±0.19 mm. The width of the maxillary dentition arch was expanded significantly in the EaT group compared with that in the other four groups. The width of the maxillary dentition arch in the EtR group was expanded significantly more than that in the NC and ExR groups.

### Midpalatal suture changes

**Figure 2:**
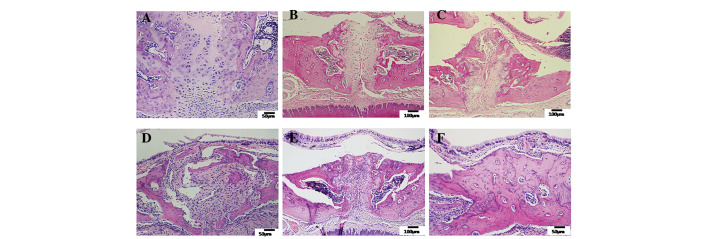
Palatal shelf structure observed after hematoxylin and eosin staining. BMMC transplantation accelerated bone remodeling through transferring endochondral bone formation into intramembraneous bone formation. Images from the (A) normal (BC group) rats and the rats of the (B) NC, (C) Exp, (D) EaT, (E) ExR and (F) EtR groups. BMMC, bone marrow mononuclear cell; BC, blank control; NC, non-expansion control; Exp, expansion; EaT, expansion and transplantation; ExR, expansion and relapse; EtR, expansion, transplantation and relapse.

A complete palatal shelf structure revealed by hematoxylin and eosin staining in a normal SD rat is shown in Fig. 2A. The chondrocytes on each side of the suture gradually rose to mature laterally toward bone-marrow-like cavities and the compact bone of the maxilla. Two weeks after an incision was made in the midpalatal suture in the NC group (Fig. 2B), the cartilage at the midpalatal suture remained separated. Some of the mesenchymal spindle cells had crawled along the midline of the disconnected surface of the midpalatal suture area. In the Exp group (Fig. 2C), the chondrocytes were arranged more laterally with mesenchymal cells migrating and bridging one-third of the midpalatal suture 2 weeks later. In the ExR group (Fig. 2E), the midpalatal suture was almost bridged by a large quantity of mononuclear cells. The chondrocytes remained evident and involved in the endochondral-type bone formation. The palatine vessels and nerve bundles in these groups were stretched, and the compact bone on both sides of the midpalatal suture did not exhibit evident changes.

The morphology of the midpalatal suture changed significantly in the EaT group (Fig. 2D). All of the chondrocytes disappeared and were replaced by fibrous-like tissues, which contained many mesenchymal cells migrating from the submucosal tissue. The submucosal layer became thicker and a large number of mesenchymal cells entered into the surrounding compact palatal bone and transformed it into trabecular bone. In the EtR group (Fig. 2F), the suture and surrounding tissues had organized from an earlier cancellous form (Fig. 2D) into a mature compact structure with several small vessels.

### Elevated osteocalcin expression in the EaT group

**Figure 3:**
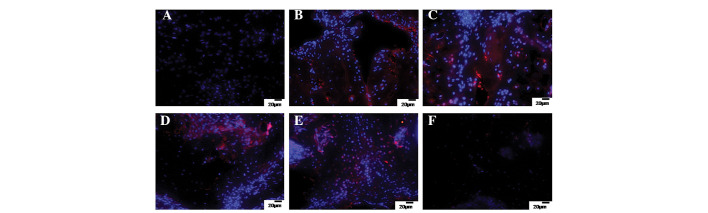
Immunofluorescent staining results of osteocalcin in the midpalatal suture. (A) Normal (BC group) rat. (B) NC group; the chondrocytes at either side of the midpalatal suture had moderate immunoreactivities. (C) Exp group; osteocalcin expression was observed in the chondrocytes. (D) EaT group; strong osteocalcin expression was detected in most of the spindle cells and extracellular matrix in the midpalatal suture area. (E) ExR group; moderate osteocalcin expression was observed in the migrated mononuclear cells whereas strong signals could be detected in the chondrocytes laterally to the suture; (F) EtR group; no osteocalcin expression observed when the suture and the palatal bone transformed into a whole mature compact structure. BC, blank control; NC, non-expansion control; Exp, expansion; EaT, expansion and transplantation; ExR, expansion and relapse; EtR, expansion, transplantation and relapse.

Fig. 3 exhibits the immunofluorescent staining results of osteocalcin. In normal SD rats (BC group), no osteocalcin expression was found in the midpalatal suture and surrounding bone (Fig. 3A). In the NC (Fig. 3B), Exp (Fig. 3C), and ExR (Fig. 3E) groups, osteocalcin expression was observed in the chondrocytes adjacent to the suture. The mature chondrocytes toward the bone marrow cavity exhibited an increased expression, indicating endochondral bone formation. The cells migrating from the mucosa were not positive for osteocalcin after 2 weeks (Fig. 3C) but exhibited moderate signals during the relapse period (Fig. 3E). Heterologous BMMC transplantation in the EaT group (Fig. 3D) resulted in a large quantity of spindle cells with strong osteocalcin expression, and when presented in the fibrous-like tissue at the midline of the midpalatal suture area, they appeared as intramembranous bone formation. However, no osteocalcin expression was observed when the suture and palatal bone transformed into a mature compact structure in the EtR group (Fig. 3F).

### Osteogenic differentiation of BMMCs transplanted into the midpalatal suture

**Figure 4:**
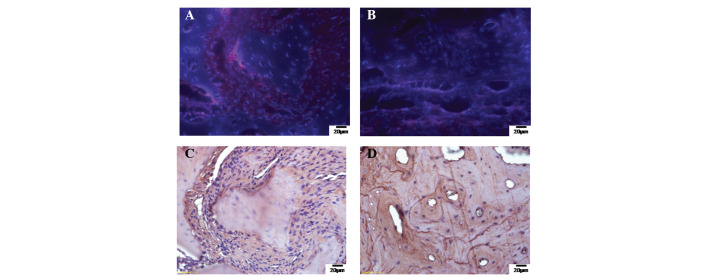
Co-localization of BrdU and osteocalcin expression in the midpalatal suture. Positive signals due to labeling with BrdU in the (A) EaT and (B) EtR groups revealed that transplanted BMMCs survived and appeared in the fibrous-like tissues. Co-expression was observed in certain (C) proliferating cells adjacent to the palatal bone of the EaT group and (D) osteocytes in the newly forming bone of the EtR group. BrdU, bromodeoxyuridine; EaT, expansion and transplantation; EtR, expansion, transplantation and relapse; BMMC, bone marrow mononuclear cell.

The transplanted BMMCs labeled with BrdU were tracked in the fibrous-like tissues at the suture of the EaT group (Fig. 4A) and at the junction of the bone and submucosal tissue in the EtR group (Fig. 4B). Double immunostaining showed that in the EaT group (Fig. 4C), certain proliferating cells were positive for both BrdU and osteocalcin; the cells were adjacent to the palatal bone in the midpalatal suture. In the EtR group (Fig. 4D), certain osteocytes in the newly forming bone showed co-localization of BrdU and osteocalcin, indicating that the transplanted BMMCs were involved in the new bone formation induced by mechanical expansion.

### RT-qPCR results of RANKL and OPG gene expression

Table II shows the ratio of RANKL/OPG expression. A significant reduction in the RANKL/OPG ratio directly resulting from inhibition of RANKL expression was observed in the EaT group and indicated bone formation as the principal activity. The maximal RANKL/OPG ratio appeared in the ExR group, indicating that bone resorption remained evident after 4 weeks of midpalatal expansion.

## Discussion

Orthodontists often use midpalatal expansion to treat malocclusion caused by a narrow maxillary dental arch. With the aim of accelerating the speed of palatal remodeling, the present study investigated the transplantation of BMMCs into the intraoral muscles of rats subjected to midpalatal expansion. The cartilage adjacent to the suture was replaced by a large quantity of fibrous tissue after 2 weeks of expansion. This result may explain why the distance between the molars increased to the greatest extent in the EaT group, as a consequence of the reduction in skeletal resistance. Heterologous BMMC transplantation significantly reduced the extent of relapse 4 weeks after expansion. Compared with the endochondral bone formation 2 weeks after expansion without BMMC transplantation, intramembranous bone formation was evident in the BMMC transplantation group. Although intramembranous bone formation appeared in the expansion group 4 weeks later, BMMC transplantation promoted the remodeling activity and completed midpalatal suture reconstruction with compact bone.

A number of studies have shown the potential benefit of bone marrow-derived mesenchymal stem cell transplantation in the enhancement of bone repair (21–23). Certain researchers have attributed osteopenia/osteoporosis to allogeneic stem cell transplantation (24), whereas others have argued that cultured bone marrow stem cells help reduce the severity of osteoporosis and increase bone mineral density by decreasing markers of resorption (25). In the present study, BMMC transplantation in midpalatal sutures accelerated the bone remodeling process over 4 weeks through absorption into the suture cartilage. With invasion by a number of mesenchymal cells derived from local stromal cell populations, transplanted BMMCs healed the dissected and expanded midpalatal suture with completely ossified bone.

Although emerging evidence suggests that bone marrow mesenchymal stem cells may have utility for skeletal repair, the related mechanisms remain unclear. The present study indicates that these cells were involved directly in bone remodeling through migration, proliferation and differentiation into osteoblasts with osteocalcin expression. Another study has also reported that BMMC transplantation is able to enhance bone formation indirectly by secreting paracrine factors and recruiting perivascular stem cells to healing wounds (12).

The maintenance of bone structure is usually considered as a process in which a balance between osteoclastic bone resorption and osteoblastic bone formation is achieved to fulfill its function. The OPG-RANK-RANKL signaling pathway is a key regulator of osteoblasts and osteoclasts to ensure normal bone turnover (26). An increasing RANKL/OPG ratio indicates an imbalance that favors resorption over weight formation during bone remodeling (20). Kon *et al* (27) demonstrated that RANKL and OPG are involved in fracture healing and the regulation of endochondral resorption and bone remodeling. Bone loss is a common complication following allogeneic stem cell transplantation, possibly through an increased RANKL/OPG ratio in the bone microenvironment, which results in the promotion of bone resorption activities (28). In the present study, the temporal-spatial expression patterns of RANKL and OPG in the midpalatal expansion groups (Exp and ExR) are in agreement with those reported in the study by Zhu (29).

In the present study, BMMC transplantation significantly reduced the RANKL/OPG ratio 2 weeks after midpalatal expansion by inhibiting RANKL expression, indicating that intensive osteogenic activities occurred prior to this time in the midpalatal expansion group without BMMCs. The RANKL/OPG ratio recovered in the EtR group, suggesting that osteoblast and osteoclast activities regained equilibrium 4 weeks later, during relapse. However, further studies are required to clarify the long-term effect of BMMC transplantation on the whole body.

## Figures and Tables

**Table I:** Primer sequences used for RT-qPCR.

Gene	Forward primer sequence	Reverse primer sequence	Accession number
RANKL	5′-AGCGCTTCTCAGGAGTTCCA-3′	5′-GCCGGGCCACATCGA-3′	NM_057149
OPG	5′-GCTGGCACACGAGTGATGAA-3′	5′-CGGTCTGCAGTTCCTTGCA-3′	U94330.1
β-actin	5′-CTTCAACACCCCAGCCATGT-3′	5′-CAGAGGCATACAGGGACAACA C-3′	NM_031144.3

RT-qPCR, reverse transcription-quantitative polymerase chain reaction; RANKL, receptor activator of nuclear factor κB ligand; OPG, osteoprotegerin.

**Table II:** RT-qPCR analysis of RANKL/OPG expression in midpalatal expansion (mean ± SD).

Group	RANKL	OPG	RANKL/OPG ratio
NC	1.20±0.12^a^	1.59±0.56	1.12±0.52
Exp	1.03±0.32^a^	0.48±0.26	2.04±0.75
EaT	0.00±0.00	0.31±0.19	0.02±0.01
ExR	0.69±0.28^a^	0.41±0.23	2.89±1.00
EtR	0.29±0.13	1.24±0.70	0.99±0.58

a P<0.05 compared with the EaT group.

RT-qPCR, reverse transcription-quantitative polymerase chain reaction; RANKL, receptor activator of nuclear factor κB ligand; OPG, osteoprotegerin; NC, non-expansion control; Exp, expansion; EaT, expansion and transplantation; ExR, expansion and relapse; EtR, expansion, transplantation and relapse.

## References

[1] de Gurgel JA, Malmström MF, Pinzan-Vercelino CR (2012). Ossification of the midpalatal suture after surgically assisted rapid maxillary expansion. Eur J Orthod.

[2] Ahrari F, Eslami N (2011). Nonsurgical treatment of maxillary deficiency using tongue guard appliance: a case report. J Dent Res Dent Clin Dent Prospects.

[3] Verstraaten J, Kuijpers-Jagtman AM, Mommaerts MY (2010). A systematic review of the effects of bone-borne surgical assisted rapid maxillary expansion. J Craniomaxillofac Surg.

[4] Petrick S, Hothan T, Hietschold V (2011). Bone density of the midpalatal suture 7 months after surgically assisted rapid palatal expansion in adults. Am J Orthod Dentofacial Orthop.

[5] Wertz RA (1970). Skeletal and dental changes accompanying rapid midpalatal suture opening. Am J Orthod.

[6] Chung CH, Font B (2004). Skeletal and dental changes in the sagittal, vertical, and transverse dimensions after rapid palatal expansion. Am J Orthod Dentofacial Orthop.

[7] Lines PA (1975). Adult rapid maxillary expansion with corticotomy. Am J Orthod.

[8] Lagravere MO, Major PW, Flores-Mir C (2005). Long-term dental arch changes after rapid maxillary expansion treatment: a systematic review. Angle Orthod.

[9] Vardimon AD, Brosh T, Spiegler A, Lieberman M, Pitaru S (1998). Rapid palatal expansion: Part 1. Mineralization pattern of the midpalatal suture in cats. Am J Orthod Dentofacial Orthop.

[10] Bianchi A, Amadori S, Pironi M, Marchetti C (2009). Maxillary expansion and stability in the orthodontic-surgical treatment of skeletal anterior open bites. Prog Orthod.

[11] Oztürk F, Babacan H, Inan S, Gümüş C (2011). Effects of bisphosphonates on sutural bone formation and relapse: A histologic and immunohistochemical study. Am J Orthod Dentofacial Orthop.

[12] Sawada M, Shimizu N (1996). Stimulation of bone formation in the expanding mid-palatal suture by transforming growth factor-beta 1 in the rat. Eur J Orthod.

[13] Khosla S, Westendorf JJ, Mödder UI (2010). Concise review: Insights from normal bone remodeling and stem cell-based therapies for bone repair. Stem Cells.

[14] Li Z, Liao W, Zhao Q (2013). Angiogenesis and bone regeneration by allogeneic mesenchymal stem cell intravenous transplantation in rabbit model of avascular necrotic femoral head. J Surg Res.

[15] Li F, Wang X, Niyibizi C (2010). Bone marrow stromal cells contribute to bone formation following infusion into femoral cavities of a mouse model of osteogenesis imperfecta. Bone.

[16] Quarto R, Thomas D, Liang CT (1995). Bone progenitor cell deficits and the age-associated decline in bone repair capacity. Calcif Tissue Int.

[17] Liang CT, Barnes J, Seedor JG (1992). Impaired bone activity in aged rats: alterations at the cellular and molecular levels. Bone.

[18] Lee K, Sugiyama H, Imoto S, Tanne K (2001). Effects of bisphosphonate on the remodeling of rat sagittal suture after rapid expansion. Angle Orthod.

[19] Kobayashi ET, Hashimoto F, Kobayashi Y (1999). Force-induced rapid changes in cell fate at midpalatal suture cartilage of growing rats. J Dent Res.

[20] Pérez-Sayáns M, Somoza-Martin JM, Barros-Angueira F, Rey JM, García-García A (2010). RANK/RANKL/OPG role in distraction osteogenesis. Oral Surg Oral Med Oral Pathol Oral Radiol Endod.

[21] Hernigou P, Poignard A, Beaujean F, Rouard H (2005). Percutaneous autologous bone-marrow grafting for nonunions. Influence of the number and concentration of progenitor cells. J Bone Joint Surg Am.

[22] Ma JT, Yu M, Zhang MC (2009). Clinical observation on percutaneous autologous bone marrow grafting for treatment of fracture nonunion. Zhongguo Gu Shang.

[23] Hatzokos I, Stavridis SI, Iosifidou E, Karataglis D, Christodoulou A (2011). Autologous bone marrow grafting combined with demineralized bone matrix improves consolidation of docking site after distraction osteogenesis. J Bone Joint Surg Am.

[24] Yao S, McCarthy PL, Dunford LM (2008). High prevalence of early-onset osteopenia/osteoporosis after allogeneic stem cell transplantation and improvement after bisphosphonate therapy. Bone Marrow Transplant.

[25] (2012). The treatment of osteoporosis through transplantation of bone marrow stem cells in the experiments performed on rats. Georgian Med News.

[26] Trouvin AP, Goëb V (2010). Receptor activator of nuclear factor-kappaB ligand and osteoprotegerin: maintaining the balance to prevent bone loss. Clin Interv Aging.

[27] Kon T, Cho TJ, Aizawa T (2001). Expression of osteoprotegerin, receptor activator of NF-kappaB ligand (osteoprotegerin ligand) and related proinflammatory cytokines during fracture healing. J Bone Miner Res.

[28] Ricci P, Tauchmanova L, Risitano AM (2006). Imbalance of the osteoprotegerin/RANKL ratio in bone marrow microenvironment after allogeneic hemopoietic stem cell transplantation. Transplantation.

[29] Zhu WQ, Wang X, Wang XX, Wang ZY (2007). Temporal and spatial expression of osteoprotegerin and receptor activator of nuclear factor -kappaB ligand during mandibular distraction in rats. J Craniomaxillofac Surg.

